# Quantifying the two-state facilitated diffusion model of protein–DNA interactions

**DOI:** 10.1093/nar/gkz308

**Published:** 2019-05-02

**Authors:** Itai Leven, Yaakov Levy

**Affiliations:** Department of Structural Biology, Weizmann Institute of Science, Rehovot 76100, Israel

## Abstract

The current report extends the facilitated diffusion model to account for conflict between the search and recognition binding modes adopted by DNA-binding proteins (DBPs) as they search DNA and subsequently recognize and bind to their specific binding site. The speed of the search dynamics is governed by the energetic ruggedness of the protein–DNA landscape, whereas the rate for the recognition process is mostly dictated by the free energy barrier for the transition between the DBP’s search and recognition binding modes. We show that these two modes are negatively coupled, such that fast 1D sliding and rapid target site recognition probabilities are unlikely to coexist. Thus, a tradeoff occurs between optimizing the timescales for finding and binding the target site. We find that these two kinetic properties can be balanced to produce a fast timescale for the total target search and recognition process by optimizing frustration. Quantification of the facilitated diffusion model by including a frustration term enables it to explain several experimental observations concerning search and recognition speeds. The extended model captures experimental estimate of the energetic ruggedness of the protein–DNA landscape and predicts how various molecular properties of protein–DNA binding affect recognition kinetics. Particularly, point mutations may change the frustration and so affect protein association with DNA, thus providing a means to modulate protein–DNA affinity by manipulating the protein’s association or dissociation reactions.

## INTRODUCTION

DNA-binding proteins (DBPs) possess a remarkably efficient ability to search and recognize their specific binding sites embedded within the genomic DNA. Early experiments showed that DBPs can find their target site at a rate two-orders of magnitude faster than the 3D diffusion limit (1). Following these results, it was suggested that DBPs accelerate the search for their target site via a mechanism of facilitated diffusion in which 1D diffusion alternates with 3D diffusion during the search (2,3). 1D diffusion itself comprises two distinct search modes, sliding and hopping, which differ in the degree to which translocation and rotation along the DNA are coupled as well as in the dependency of their corresponding diffusion coefficients on salt concentration (4–6). The understanding that DBP search of DNA proceeds through a combination of diffusions in different dimensional spaces is referred to as the facilitated diffusion model. This model is well supported by numerous experimental and theoretical studies and is able to explain the high target association rates observed *in vitro* and in the cell (7–13).

The biophysical characteristics of DNA search may depend on the molecular properties of the searching proteins. For example, the dimensions of the protein, its oligomeric state, electrostatic potential and degree of flexibility may affect the relative usage of the different search modes (14–19). The DNA sequence may also affect search speed by producing different DNA geometries and consequently affecting the protein’s ability to interact with the DNA major groove and thus its ability to perform coupled rotation-translation diffusion (20,21). The energy landscape for 1D diffusion along DNA may also be affected by the DNA sequence, which can affect the ruggedness of the potential energy landscape and, consequently, friction in protein–DNA interactions (9,22,23).

The 1D diffusion coefficient of proteins on DNA has been expressed as *D* = *G*(*T,R,η*)·*F*(*σ*), where *G* is a term that depends on the temperature, *T*, the size of the protein, *R*, and the viscosity of the solution, η (10). *F* is a term that represents the ruggedness of the potential energy landscape. Assuming that the ruggedness of the potential energy follows a Gaussian distribution, then *F*(ϵ) = exp[–(*σ/k*_B_*T*)^2^], where *σ* denotes the variance in the protein–DNA sliding potential and is related to the average energetic barrier for sliding (9,10,23). It was estimated that the ruggedness of the protein–DNA landscape must be low (*σ* < 2*k*_B_*T*) to achieve reasonable association rates (9).

Although many studies aimed to biophysically characterize the mechanism of facilitated diffusion that proteins adopt when searching for their target sites (2,7,24–31), it is clear that the kinetics of many protein–DNA recognition interactions does not depend solely on the search speed. Recognition requires not only finding the target site, but also specifically binding to it. The time-scale for specific binding may affect the kinetics of protein–DNA recognition because finding the site does not guarantee immediate binding. The discrepancy between finding and binding the target site is related to the different types of interactions used for the search and recognition modes. In the search mode (designated here as the *S* state), a DBP interacts with DNA non-specifically through mostly electrostatic interactions between positively charged protein residues and negatively charged phosphates on the DNA backbone (32,33). Specific binding upon reaching the target site requires the protein to switch to its recognition mode (designated here as *R* state) by forming sequence specific contacts with the DNA, supported by hydrogen bonding between the protein residues and the DNA bases (34). Thus, the full search kinetics involves not only searching in the non-specific binding mode, *S*, but also switching to the specific binding mode, *R*, upon recognition of the cognate binding site (11,23,35–39). It was argued that the existence of the *S* and *R* binding modes is necessary to solve an apparent conflict between speed and stability whereby the conditions for fast search (which requires *σ* < 2*k*_B_*T*) are incompatible with stable protein–DNA interactions (which requires *σ* > 5*k*_B_*T*) (9,23). This two-state model is supported by various experimental approaches (including X-ray crystallography, NMR and single-molecule techniques) indicating that numerous DBPs adopt different conformations for their specific versus non-specific interactions with DNA (8,14,23,40–44). The existence of two states can also be inferred from the low roughness for sliding that was found for various proteins (10,45), which is unlikely for the *R* states.

The existence of the *S* and *R* binding modes suggests the presence of an energetic barrier governing the transition between them and therefore a separation of time scale between finding and binding the target site. This implication is supported by single-molecule experiments conducted in living cells on the lac repressor, which displays facilitated diffusion characterized by a low sliding energy barrier of *ϵ* ∼ 1.0 *k*_B_*T* and a scanning length of $\ 45 \pm 10$ base pairs for each 1D search round (8). Surprisingly, the lac repressor was found to slide numerous times over its promoter before target recognition is achieved, which reflects the existence of a barrier that needs to be overcome for specific recognition to occur.

Despite being insightful, the two-state model for protein–DNA recognition is still not quantitative. In particular, the interplay between the speed of sliding (via the *S* mode) and the recognition rate (transition from the *S* to *R* mode) is unclear. While some crystal structures of protein–DNA complexes reveal conformational changes associated with the transition from non-specific to specific binding (32,33,46), other structures indicate high similarity between the non-specific and the specific binding modes (47,48). The latter scenario describes reactions with much smaller barriers for recognition in comparison with the former scenario. One may therefore ask: what are the kinetic consequences for sliding dynamics and what is the overall kinetics when the barriers between the *S* and *R* binding modes are small? Furthermore, is the rate limiting step in protein–DNA recognition the search kinetics or the transition barrier from *S* to *R*?

**Figure 1.**
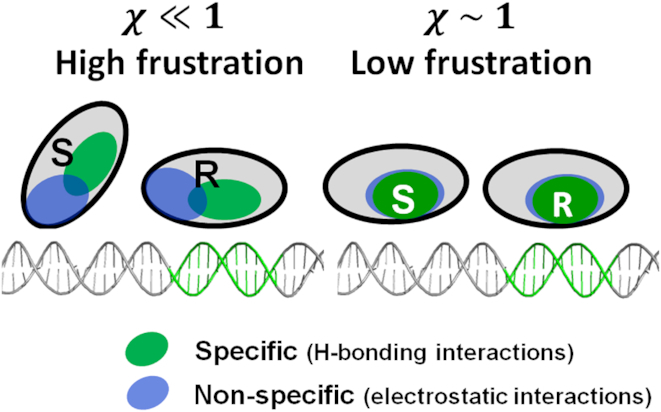
Schematic representation of the search and recognition modes of a DNA-binding protein on DNA. The degree of similarity between the search and recognition binding modes (designated as *S* and *R*, respectively) is governed by the extent of overlap between the surface patches the DNA-binding protein uses to interact with non-specific and specific DNA residues. Non-specific binding is dictated by electrostatic interactions (indicated by the blue ellipse), whereas specific binding is dictated by a set of hydrogen bonds that can be identified from the X-ray structures (indicated by the green ellipse). The overlap between the *S* and *R* binding modes is quantified by the similarity index, *χ*. A smaller overlap between the non-specific and specific patches (i.e. *χ* << 1) corresponds to high frustration and suggests that the protein needs to undergo a conformational change (which potentially involves a change in the DNA conformation as well) to make the *S → R* transition and so bind its DNA target site (left). A high value of *χ* (∼ 1) reflects less frustration between the *S* and *R* states and suggests that these two states are very similar (right).

In recent studies, we introduced the concept of molecular frustration denoted χ between the non-specific and specific protein–DNA binding modes (40,41,49). Frustration is quantified as the degree of overlap between the protein surface patches that are used for the *S* and *R* binding modes. For a given protein, the *S* mode is represented by the largest positively charged patch on the protein surface and the *R* mode is given by its X-ray structure. Greater frustration corresponds to a smaller overlap between the *S* and *R* binding modes (Figure 1). Frustration between the protein residues forming specific versus non-specific contacts with the DNA creates means that the two binding modes represent different energetic and conformational states, so creating the two-state model (40,41). It was shown that numerous DBPs, and particularly enzymes, have a medium to high degree of frustration between their *S* and *R* binding modes. Furthermore, coarse-grained molecular dynamics simulations qualitatively showed that frustration strongly influences the kinetics of the protein–DNA search process, such that high (low) frustration is associated with fast (slow) sliding but a poor (high) target recognition probability (41).

In this paper, we show how frustration links together two of the most prominent aspects of the protein–DNA recognition process, namely, the kinetics of finding the target site via the facilitated diffusion mechanism and the kinetics of binding the target, which depend on the target recognition probability. We extend Slutsky and Mirny's (9) facilitated diffusion theory by introducing the concept of frustration between the non-specific and specific binding modes and its effect on DNA recognition kinetics. Utilizing the new theoretical model, we elucidate and enable quantification of various factors influencing protein–DNA recognition kinetics.

## MATERIALS AND METHODS

### Theoretical model

We study the kinetics of protein–DNA recognition by building on Slutsky and Mirny (9,50) facilitated diffusion theory by introducing explicit terms for the *S* to *R* transition. In the framework of this theory, a single DBP searches for its target site on a long DNA of *M* bps through rounds of 1D and 3D diffusion. The mean durations of the 1D and 3D diffusion are *τ*_1D_ and *τ*_3D_, respectively. The number of 1D and 3D rounds needed to find the target site can be estimated as *M*/<*n*>, where <*n*> is the mean number of sites scanned in each 1D round (i.e. $\langle n \rangle \ = \ 2\ \sqrt {{D_{1D}}{\tau _{1D}}}$, where *D*_1D_ is the diffusion coefficient of sliding). The mean total search time is then:

(1) \begin{equation*} {{\boldsymbol \tau} _{\boldsymbol{s}}} = \frac{{\boldsymbol{M}}}{{\left\langle n \right\rangle }}\ \left( {{{\boldsymbol{\tau }}_{1{\boldsymbol{{\rm D}}}}} + {\tau _{3{\boldsymbol{{\rm D}}}}}} \right) \end{equation*}

The expression in Equation (1) assumes that the target is recognized (i.e. *S* to *R* transition occurs) in the first search round that visits the cognate site. However, it is possible for the DPB to miss the target site and then additional 1D and 3D rounds might be needed. Accordingly, the total search time, *τ_S_*, must be multiplied by 1/*P*_f_, where *P*_f_ is the probability of recognizing the target site and, thus, the efficiency of the search. Given that, in a single round of 1D diffusion, the protein covers ∼*n* sites and makes *n*^2^ steps, each site is revisited ∼ *n* times. Thus, the overall probability of the DPB locating the target site, once the protein associates inside a region of size ∼*n* that contains the site, is *P*_loc_ = min [1, <*n*>·*P*_f_]. The total recognition time is therefore estimated by:

(2) \begin{equation*}{\tau _R} = {\tau _S}/{P_{{\rm loc}}}\end{equation*}

The value of *P*_f_ depends on the rate, *k*_res_, to move a single step while searching using the *S* state (i.e. high *k*_res_ implicates small residence time and will reduce the probability for *S → R* transition) and the transition rate, *k_S→R_*, for switching from the *S* to the *R* state. *P*_f_ thus can be expressed by:

(3) \begin{equation*}\ {{\boldsymbol{P}}_{\boldsymbol{f}}} = \frac{{{{\boldsymbol{k}}_{{\boldsymbol{S}} \to {\boldsymbol{R}}}}}}{{{{\boldsymbol{k}}_{{\boldsymbol{S}} \to {\boldsymbol{R\ }}}} + {\rm{\ }}{{\boldsymbol{k}}_{{\boldsymbol{res}}}}}}\end{equation*}

**Figure 2.**
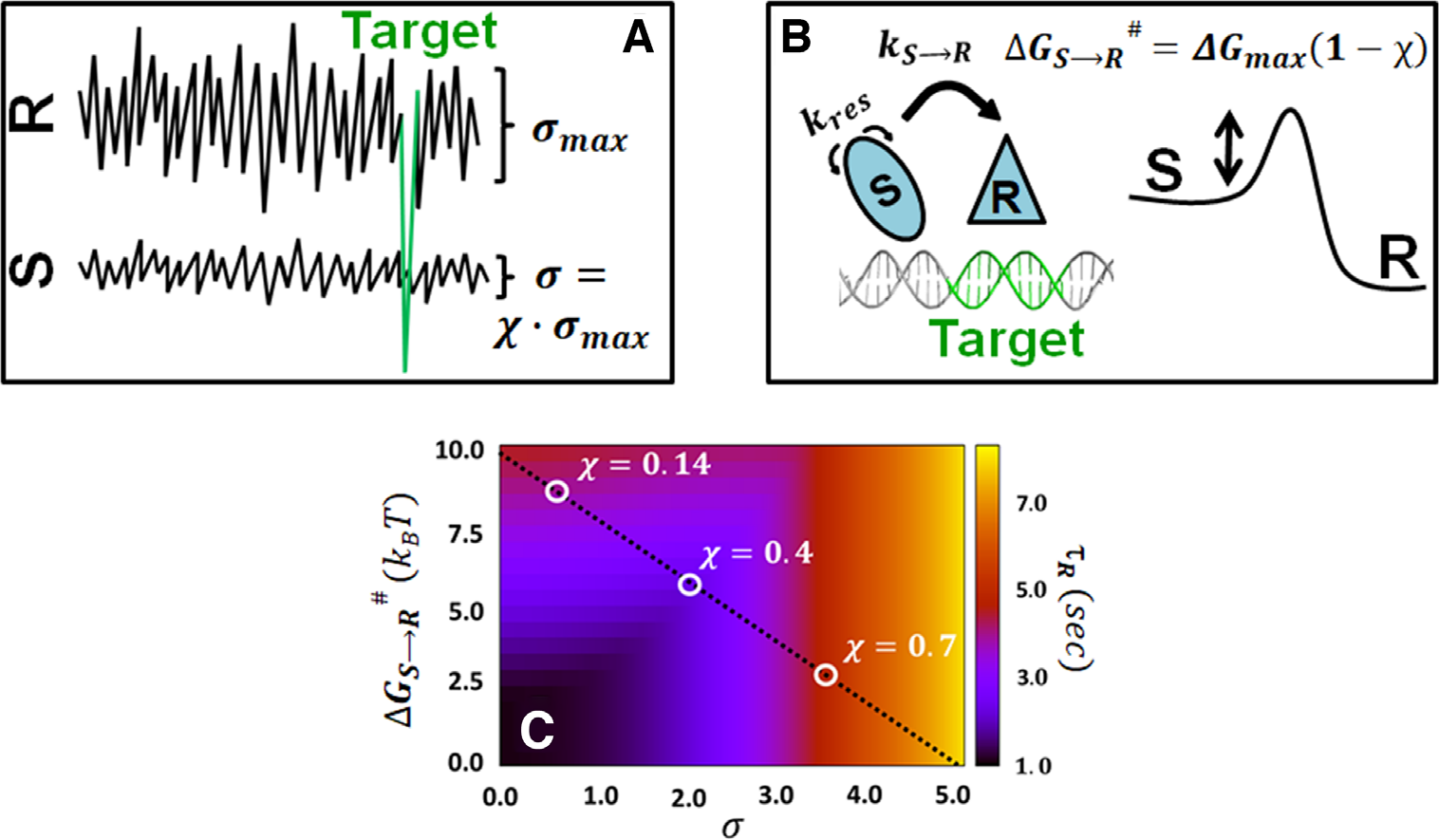
A schematic representation of the presented model for a DBP finding and binding its DNA target site. (**A**) DBP search of the DNA via 1D diffusion, during which the protein is in the *S* binding mode, is governed by the ruggedness of the protein–DNA energy landscape, *σ*. The energetic ruggedness is related to the DNA sequence and to the ability of the protein to form specific hydrogen bonds; thus, it is correlated with the similarity index, *χ*. The ruggedness and frustration are linked by the relation ${\rm{\sigma \ }} = {\rm{\ \chi }} \cdot {{\rm{\sigma }}_{{\rm{max}}}}$ where ${{\rm{\sigma }}_{{\rm{max}}}}$ is the variation of the sliding energy potential in the recognition mode. The target site (marked green) is illustrated by a deep energy well in the recognition model. (**B**) Schematic presentation of the transition from the search (*S*) to the recognition (*R*) mode at the target site with a typical transition rate of *k_S→R_* while having to cross an energy barrier of $\Delta G_{s \to R}^\ddagger$. The term *τ*_res_ ( = *k*_res_^−1^) is the average time the protein spends on one base pair during sliding. The free energy barrier $\Delta G_{s \to R}^\ddagger$ depends on the similarity index through the relation $\Delta G_{s \to R}^\ddagger$ = Δ*G*_max_(1 − *χ*), where Δ*G*_max_ represents a maximal barrier for highly frustrated protein–DNA binding. (**C**) The total time to recognize the target site, *τ*_R_, is affected by the time scale of both 1D diffusion and the transition from *S* to *R*. These two process have opposite dependencies on the similarity index. Accordingly, σ and $\Delta G_{s \to R}^\ddagger$ are negatively correlated. The dotted line expresses the values of $\Delta G_{s \to R}^\ddagger$ and of ${\rm{\sigma }}$ when they are linked through frustration according to the proposed model, and various ${\rm{\chi }}$ values are marked. The total recognition times for different values of *χ* and $\Delta G_{s \to R}^\ddagger$ are indicated by the color bar. This plot was calculated for Δ*G*_max_, *σ*_max_ and *E*_ns_ of 10, 5 and 9*k*_B_*T*, respectively.

The transition rate, ${k_{S \to R}}$, is dictated by the energy barrier $\Delta G_{s \to R}^\ddagger$ between the two binding modes (Figure 2):

(4) \begin{equation*}{{\boldsymbol{k}}_{{\boldsymbol{S}} \to {\boldsymbol{R}}}} = \frac{1}{{{{\boldsymbol{\tau }}_0}}}{\rm{\ }} \cdot {\boldsymbol{{\rm exp}}}\left( { - \frac{{\Delta G_{s \to R}^\ddagger }}{{{{\boldsymbol{k}}_{\boldsymbol{B}}}{\boldsymbol{T}}}}} \right)\end{equation*}

Where τ_0_ represents the characteristic time to move between non-specific site. It is suggested that for very small energy barrier (i.e. $\Delta G_{s \to R}^\ddagger$ ∼0), the kinetics of target recognition will be dictated by the diffusion speed. We can define ${k_{res}}$ as the inverse of the average time the protein spends on a given DNA site during 1D sliding (i.e. *k*_res_ = *τ*_res_^−1^, being the inverse of the residence time in the *S* state and is obtained by having *n* = 1 in the relation $\langle n \rangle \ = \ 2\ \sqrt {{D_{1{\rm D}}}{\tau _{1{\rm D}}}}$*)* (Figure 2B):

(5) \begin{equation*}{{\boldsymbol{k}}_{{\boldsymbol{{\rm res}}}}} = {\rm{\ }}4{{\boldsymbol{D}}_{1{{\bf D}}}}/B{P^2}\end{equation*}

The diffusion coefficient for protein sliding on a rugged DNA potential energy surface is expressed by:

(6) \begin{equation*}{{\boldsymbol{D}}_{1{\boldsymbol{{\rm D}}}}} = \frac{{{\boldsymbol{B}}{{\boldsymbol{P}}^2}}}{{{{\boldsymbol{\tau }}_0}}}{\boldsymbol{\ {\rm exp}}}\left( { - {{\left( {\frac{{\boldsymbol{\sigma }}}{{{{\boldsymbol{k}}_{\boldsymbol{{\rm B}}}}{\boldsymbol{T}}}}} \right)}^2}} \right)\end{equation*}

Where ${\tau _0}$ expresses the typical time it takes the protein to hop to a neighboring site. For a protein undergoing spin coupled diffusion (51):

(7) \begin{equation*}\ {{\boldsymbol{\tau }}_0} = \frac{{\left( {6{\boldsymbol{\pi \eta R}} + {{\left( {\frac{{2{\boldsymbol{\pi }}}}{{10{{\bf BP}}}}} \right)}^2}\left( {8{\boldsymbol{\pi \eta }}{{\boldsymbol{R}}^3} + 6{\boldsymbol{\pi \eta R}}{{\boldsymbol{R}}_{{\boldsymbol{OC}}}}^2} \right)} \right){\boldsymbol{B}}{{\boldsymbol{P}}^2}}}{{{{\boldsymbol{k}}_{\boldsymbol{{\rm B}}}}{\boldsymbol{T}}}}\ \end{equation*}

Where $R$ is the protein’s radius, ${R_{OC}}$ is the distance between the center of mass of the protein and the DNA, BP is the distance between two base pairs along the DNA axis and $\eta$ is the solution viscosity. In our model, we assumed ${R_{OC}} = R$ and a protein radius of$\ R\ = \ 3\ {\rm nm}$. The average amount of time the protein stays bound to DNA for each 1D search round is dependent on the non-specific protein–DNA binding strength, which is given by the inverse of the dissociation rate, *k*_off_, of the protein from non-specific DNA site obtaining:

(8) \begin{equation*}\ {{\boldsymbol{k}}_{{\boldsymbol{{\rm off}}}}}^{ - 1} = {\boldsymbol{\ }}{{\boldsymbol{\tau }}_{1{\boldsymbol{D}}}} = {{\boldsymbol{\tau }}_0}{\boldsymbol{\ {\rm exp}}}\left( {\frac{{{{\boldsymbol{E}}_{{\boldsymbol{{\rm ns}}}}}}}{{{{\boldsymbol{k}}_{\boldsymbol{{\rm B}}}}{\boldsymbol{T}}}} + \frac{1}{2}{{\left( {\frac{{\boldsymbol{\sigma }}}{{{{\boldsymbol{k}}_{\boldsymbol{{\rm B}}}}{\boldsymbol{T}}}}} \right)}^2}} \right)\end{equation*}

Where ${E_{{\rm ns}}}$ denotes the non-specific binding energy and is mostly governed by the electrostatic interactions between the positively charged residues and the negatively charged backbone of the DNA. The energetic contribution of state *S* may include a contribution from the formation of semi-specific interactions between the protein and sequences that share some similarity to the target site. The formation of such occasional hydrogen bonds that depend on the DNA sequence is captured by the roughness of the protein–DNA energy landscape, *σ* (Figure 2A). Combining the above equations with the diffusion law, ${\boldsymbol{\bar{n}}} = {\boldsymbol{\ }}2\sqrt {{{\boldsymbol{D}}_{1{\boldsymbol{{\rm D}}}}}{{\boldsymbol{\tau }}_{1{\boldsymbol{{\rm D}}}}}}$, the *τ_R_* term can be expressed in terms of $\sigma$, ${E_{{\rm ns}}}$, $\Delta G_{s \to R}^\ddagger$, ${\tau _0}$ and $T$. We note that in our model, the transition *S → R* can take place at any DNA site although it is likely that some unknown DNA features may support the transition state of the *S → R* transition.

In this study, we argue that the roughness of the protein–DNA energy landscape for sliding, *σ*, and the energetic barrier for the transition between the search and recognition modes, $\Delta G_{s \to R}^\ddagger$, are linked. For example, a protein having a high probability of interacting with semi-specific sites while in the *S* state is expected to transition faster from state *S* to *R*, simply because the *S* state already shares some similarity to the *R* state even prior to the transition. Accordingly, *σ* and $\Delta G_{s \to R}^\ddagger$ are expected to be anti-correlated. Namely, the *S* and *R* states are linked and the relationship between them can be quantified by the degree of frustration. The molecular frustration between the *S* and *R* binding modes can be quantified through the degree of overlap between the non-specific and specific binding patches on the DBP (Figures 1 and 2) (40,41). As the degree of overlap between the *S* and *R* binding modes (also known as the similarity index, *χ*) increases, additional residues may interact with DNA; thus *σ* increases. To model the relationship between $\Delta G_{s \to R}^\ddagger$ and *χ*, we use a coarse-grained model to simulate the rate of binding a target site for a set of six DBPs having varying *χ* values. The kinetics of recognizing the binding site can be estimated to depend exponentially on *χ* (see Supplementary Data). We therefore define linear relationships for *σ* and $\Delta G_{s \to R}^\ddagger$ with the overlap or similarity index *χ* and obtain:

(9) \begin{equation*}{\boldsymbol{\sigma \ }} = {\rm{\ }}{\boldsymbol{\chi }} \cdot {{\boldsymbol{\sigma }}_{{\boldsymbol{{\rm max}}}}}\end{equation*}

(10) \begin{equation*}\Delta G_{s \to R}^\ddagger = {\rm{\ }}\Delta {{\boldsymbol{G}}_{{\boldsymbol{{\rm max}}}}}\left( {1 - {\boldsymbol{\chi }}} \right)\end{equation*}

Where ${\sigma _{{\rm max}}}$ and $\Delta {G_{{\rm max}}}$ are the maximal ruggedness and transition energy barrier, respectively. A schematic representation of the proposed model is shown in Figure 2. The maximal sliding roughness, ${\sigma _{{\rm max}}}$, corresponds to the roughness in the recognition mode, *R*, in which all the protein residues participate in specific contacts with the DNA. Sufficient stability at the target site can be achieved for ${\sigma _{{\rm max}}} \sim 5{k_{\rm B}}T$ and therefore this value is used throughout this study (9). An estimate of the value of ${\rm{\Delta }}{G_{{\rm max}}}$ is, however, less straightforward as it can be influenced by various factors, including loss of non-specific contacts and strain, such as bending and deformation upon formation of the DNA–protein complex. We therefore evaluate the influence of various ${\rm{\Delta }}{G_{{\rm max}}}$ values on the target recognition rate. We note that the search time of the target site by DBPs may be influenced by other factors not included in the current study such as roadblocks and crowders (12,13,52), the geometric properties of the searched DNA and the locations of the target genes (25,53–55), DBP’s concentration and sequence effects of the DNA (20,39,56). Furthermore, the search kinetics might be characterized not only by the mean search time but also by the full distribution of reaction times (57,58). Despite the simplicity of the model, it quantitatively highlights the tradeoff between the search and recognition kinetics.

### Calculation of the frustration between the two-state binding modes

The similarity between the *S* and *R* states is estimated as the overlap between the binding modes. The residues that interact with DNA in the *R* mode can be obtained from crystal structures and those that interact in the *S* mode are linked to a positively charged patch on the protein surface. To estimate the overlap between *S* and *R*, we first define ${\chi _i}$ for each protein residue forming specific contacts with DNA in the complex according to the equation:

(11) \begin{equation*}\ {\chi _i} = \frac{{\left( {\mathop \sum \nolimits_j {q_j} \cdot {\rm exp}\left( { - a\frac{{{r_{ij}}}}{{{r_c}}}} \right)} \right)}}{{\left( {\mathop \sum \nolimits_j {\rm exp}\left( { - a\frac{{{r_{ij}}}}{{{r_c}}}} \right)} \right)}}\end{equation*}

Where *j* denotes any protein residue closer than a cutoff distance of *r*_c_ = 8 Å to residue *i*; ${q_j}$ is the point charge of residue *j* and takes values of −1, 0 or 1; ${r_{ij}}$ is the cutoff distance between residues *i* and *j*; and $a = 5$ is an exponential decay constant. We then average all ${\chi _i}$ values of the protein to obtain its similarity index$ \chi$. The values of *χ* lie between (−1) and (+1) and depend on the parameters *a* and *r*_c_. The larger the value of *χ* (i.e. the lower the frustration) the greater the similarity between the *S* and *R* states. The similarity indices can be evaluated by considering all the positively charged residues in the structure or by selecting those that support the *S* state. The latter subset can be elucidated from coarse-grained simulations that were applied to study the sliding of proteins along DNA (4,5). We find strong correlation between the values of *χ* calculated using these two approaches.

## RESULTS

### Trade-off between sliding rate and recognition rate

**Figure 3.**
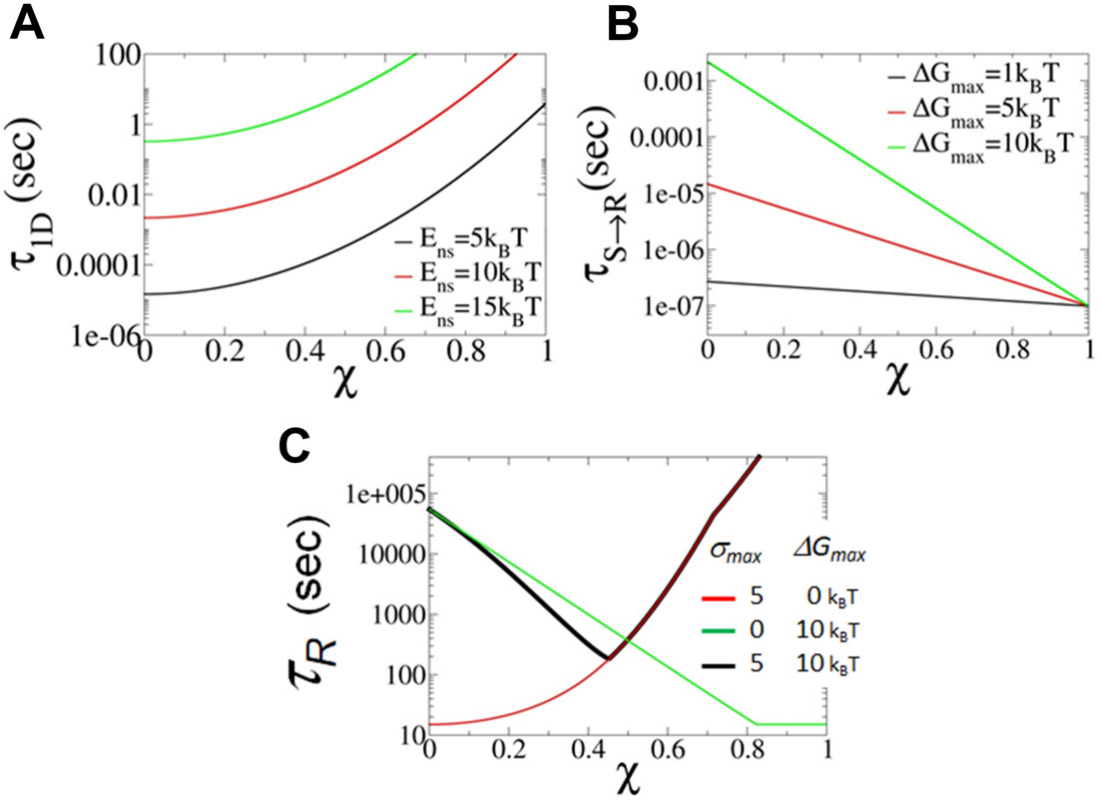
Contribution of the quantified two-state model to understanding the kinetics of DNA recognition. The existence of two states, *S* and *R*, are manifested by the similarity index, *χ*. The larger the value of *χ* (i.e. the lower the frustration) the greater the similarity of the *S* and *R* states. (**A**) The time scale of 1D diffusion, *τ*_1D_, is plotted as a function of the similarity index for three different values of non-specific DNA binding energy, *E*_ns_$,{\rm{\ }}$using a value for the free energy barrier for recognition of Δ*G*_max_ = 5*k*_B_*T*. (**B**) Transition time from *S → R* as a function of *χ* for various values of *ΔG*_max_ (*τ_S→R_* is estimated by *k_S→R_*^−1^). (**C**) Total recognition time as a function of *χ* for different values of Δ*G*_max_ and *σ*_max_ (*E*_ns_ = 5*k*_B_*T* and *M* is 5 × 10^6^).

We first address how frustration (which impedes a protein’s ability to switch from its searching mode to its recognition and binding mode) influences the different components of target recognition kinetics; namely, 1D diffusion and *S → R* transition. Such frustration is associated with a positive effect on the search kinetics by reducing the energetic roughness for sliding, *σ*, and maximizing the number of BPs scanned in each 1D search round. Figure 3A plots the elapsed time for a single 1D search round as a function of the similarity index (being negatively correlated with frustration) for three different non-specific binding energies. Overall, increased frustration (i.e. decreased $\chi$; Figure 3A) reduces the amount of time spent in each 1D search round by reducing the number of energetic traps for the searching protein on the DNA (i.e. *σ* is smaller for more frustrated interfaces, Equations 6 and 9). In addition, the 1D search time is highly sensitive to the magnitude of the non-specific binding energy, showing a four orders of magnitude difference when raising *E*_ns_ from 5 to 15 *k*_B_*T*.

Frustration also has a negative effect on the search kinetics by increasing the transition time from the *S* to *R* binding mode and thereby decreasing the probability of the protein recognizing the target DNA sequence (Equation 10). The overall transition rate for a given protein also depends on the value of Δ*G*_max_ (Figure 3B), which accounts for interaction variability in different protein–DNA complexes. For a moderate similarity index value of *χ* = 0.3, a two-order of magnitude increase in transition time is observed upon increasing the free energy barrier for recognition, $\Delta {G_{{\rm max}}}$, from $5{k_{\rm B}}T$ (red line) to $\ 10{k_{\rm B}}T$ (green line).

Figure 3C plots the total mean search time as a function of similarity index under three conditions: the two extreme search kinetics settings for a smooth sliding landscape (i.e. ${\sigma _{{\rm max}}} = \ 0$) and an immediate transition *S → R* (i.e. $\Delta \ {G_{{\rm max}}} = \ 0$). The figure shows that, for cases with low transition barriers (Figure 3C, red line), the DBP favors high frustration and exhibits fast 1D diffusion. At the other extreme, where the protein lacks sliding roughness but the transition barrier is high, the protein favors low frustration (Figure 3C, green line). In the presence of both an energy barrier and sliding roughness (Figure 3C, black line), there exists an optimal frustration value at which both sliding and target recognition proceed at an adequate speed. The optimal frustration value between the *S* and *R* states may vary for different proteins depending on their electrostatic energy to bind DNA (i.e. *E*_ns_) and the free energy barrier for recognition (i.e. Δ*G*_max_).

**Figure 4.**
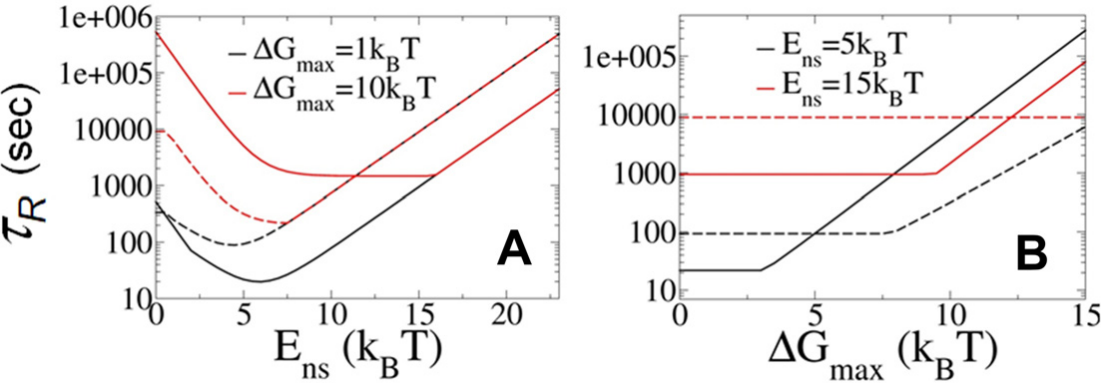
The effect of non-specific binding energy and transition barrier height on the total recognition time. (**A**) Total recognition time, *τ*_R_, as a function of non-specific binding energy, *E*_ns_, for two values of Δ*G*_max_ plotted for frustration indices *χ* = 0.2 (solid lines, high frustration) and *χ* = 0.4 (dashed lines, low frustration). (**B**) Total recognition time as a function of the maximal energy barrier for transition, Δ*G*_max_, for two *E*_ns_ values plotted using *χ* = 0.2 (solid lines) and *χ* = 0.4 (dashed lines).

To understand better how the molecular properties of the recognition process affect its kinetics, we study the relationship between the non-specific (electrostatic) binding energy (*E*_ns_), the magnitude of the transition energy barrier (Δ*G*_max_) and the total recognition time (*τ*_R_) for two different similarity index values (*χ*) as a protein shifts between the *S* and *R* states. Figure 4A plots *τ*_*R*_ as a function of *E*_ns_. Changing the value of *E*_ns_ can be viewed as equivalent to changing the salt concentration, which is well known to tune the strength of non-specific protein–DNA binding. At high non-specific binding energies, the total recognition time is long (Figure 4A). This is due to the sluggish 1D dynamics at high values of *E*_ns_ (Equation 8). At low values of *E*_ns_, 1D scanning in each round of 1D and 3D search is less efficient, as the time spent in the sliding mode is shorter and consequently the number of scanned sites in each round is smaller as $\langle n \rangle \ = {\boldsymbol{\ }}2\sqrt {{D_{1{\rm D}}}{\tau _{1{\rm D}}}}$. As <*n*> decreases, the total recognition time, *τ*_R_, becomes longer (Equation 1). The dependency of the total recognition time on *E*_ns_, as obtained from our theory, therefore supports one of the hallmarks of the facilitated diffusion model regarding the existence of an optimal combination of 1D and 3D search modes (2,4,9,24). It can be seen that proteins with a high *S* to *R* transition barrier (Figure 4A, red curves) prefer greater frustration, that is, a lower similarity index (Figure 4A, solid versus dashed lines) in order to reduce the sliding barrier and maximize the number of target binding attempts required in each 1D search round. However, proteins with lower transition barriers (black line) do not require multiple target recognition attempts and therefore prefer lower frustration, that is, a higher similarity index (Figure 4A, dashed versus solid lines). The effect of *E*_ns_ on the recognition rate has been shown experimentally; increasing the salt concentration markedly affects the association rate while the dissociation rate is hardly affected (59).

Figure 4B plots the total recognition time as a function of the maximal transition energy barrier, Δ*G*_max_. The figure shows that, for a given non-specific binding energy, *E*_ns_, the recognition timescale reduces as Δ*G*_max_ decreases until the value of Δ*G*_max_ is low and the probability for *S → R* transition is very high (i.e. *P*_f_ = 1). Figure 4B also shows that faster recognition is achieved for more frustrated proteins (lower similarity index; solid compared to dashed lines).

### Frustration is linked with the diffusion coefficient for sliding

**Figure 5.**
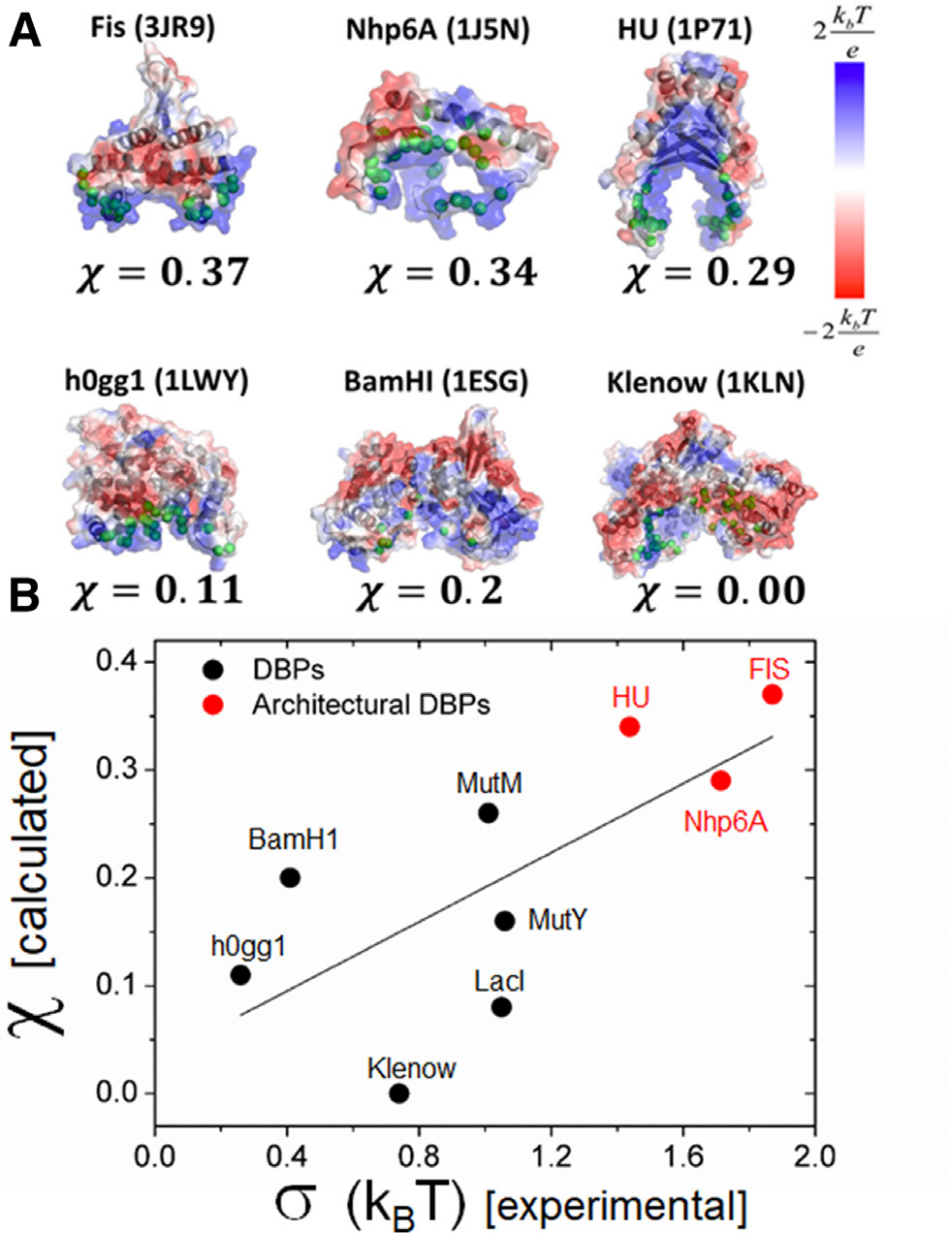
Relationship between the ruggedness of the protein-DNA energy landscape and the frustration between the *S* and *R* states. (**A**) Schematic illustration of the electrostatic potential surface (color bar on the right) and residues that are found to participate in specific contacts with the DNA (green beads) for several proteins whose linear diffusion coefficients were measured experimentally. The calculated similarity index, *χ*, is shown below each structure. (**B**) Correlation between the calculated similarity index and the experimentally estimated roughness of the energy landscape (*σ*) obtained from the linear diffusion coefficient (60). The three DNA-binding proteins (DBPs) that are marked in red were classified as architectural binding proteins. The correlation between *χ* and *σ* suggests that greater energetic barriers to sliding are related to low frustration (high *χ*) and also points to the molecular difference between architectural and non-architectural DNA-binding proteins.

Our group has previously calculated molecular frustration for a dataset of 125 DBPs on the basis of the crystal structure of their complexes with DNA and with the goal of estimating the degree of frustration for various protein structures and functions (40,41). Here, we quantify the frustration indices for nine DBPs (Equation 11) whose linear diffusion coefficient, *D*_1D_, was measured experimentally and whose crystal structures with DNA are known. Figure 5A shows pictorially the overlap between the *R* state (represented by the green spheres) and the *S* state (represented by the blue surface and corresponding to an electrostatically positive patch). As can be seen in Figure 5A, proteins with low frustration (top row) obtain high overlap (i.e. high *χ* values) and vice versa for proteins with low frustration (bottom row). The similarity index can explain the linear diffusion coefficient of different DBPs, because *χ* is linked to the roughness of the protein–DNA energy landscape, *σ*. Recently, experimental data have shown architectural binding proteins to be associated with relatively high energetic roughness compared with other DBPs (60). According to our model, we expect these proteins to be characterized by low frustration (i.e. high *χ* values). Figure 5B plots the frustration indices of these nine DBPs and the experimental $\sigma$ values, which were derived from their corresponding *D*_1D_ values. The correlation between *χ* and *σ*, shown in Figure 5B, strongly supports our model regarding the linkage between frustration and sliding (Equation 9). It is shown that, indeed, architectural binding proteins are associated with high *χ* values. We therefore hypothesize that, while transcription factors are optimized for fast search and therefore high frustration (low *χ* value), architectural binding proteins are less frustrated and diffuse more slowly, as required for their function.

### Frustration can explain the effect of mutations on recognition kinetics

The effect of point mutations on the kinetics of the *S* → *R* transition can be predicted by the frustration between these two states. Experiments measuring the association and dissociation rates of mutated proteins are routinely performed to shed light on the mechanism of protein–DNA kinetics (59,61,62). Generally, these experiments measure the binding kinetics of DBPs with short DNA oligonucleotides containing the target site and therefore they do not involve significant 1D sliding on DNA. Accordingly, we postulate that mutations that change the level of frustration will also modify the protein–DNA association rates by shifting the transition energy barrier, Δ*G_S→R_*. The transition barrier is expected to increase for larger frustration (i.e. for lower *χ*; Equation 10).

**Figure 6.**
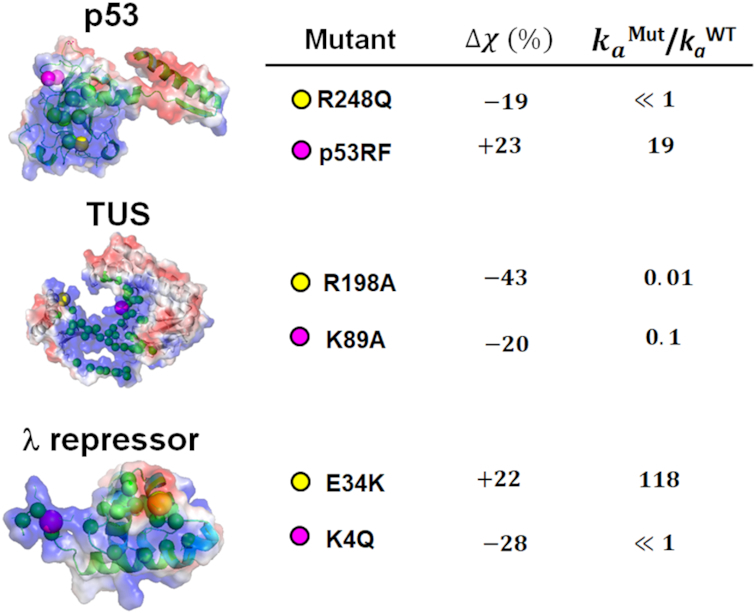
The effect of mutations on the kinetics of DNA recognition can be explained by a change in the frustration. The kinetics of binding to a specific site on DNA was analyzed for six mutants of three different proteins (P53, TUS and ${\rm{\lambda }}$ repressor proteins). The electrostatic potential of each of the three proteins is shown. In each protein, the residues that participate in specific contacts with DNA (green beads) as well as the mutated sites (yellow and magnetite beads) are shown. The mutants are analyzed in terms of the change in the similarity index, Δ*χ*, in comparison with the wild-type protein and with respect to the association rate relative to the wild-type protein, *k*_a_^Mut^/*k*_a_^WT^. Association rate values are taken from (59,66,68). The PDB codes used to calculate the similarity index: P53RF (4MZRa), P53wt (3Q05), TUS (1ECR) and ${\rm{\lambda }}$ repressor (1LMB).

To test our hypothesis, we calculate the change in the similarity index for six different mutants of the p53, TUS and $\lambda$ repressor proteins and for the corresponding wild-type proteins. Then, the experimentally measured association rates are compared to the change in *χ*. The results are summarized in Figure 6. For the p53 protein, we studied frustration in the R248Q mutant, which has been shown to be inactivate (63,64), and for the p53FG mutant, which contains two substitutions, S121F and V122G in the Loop L1 and is associated with high activation rates (65,66). The similarity index of the wild-type p53 is $\chi \ = \ 0.26$ whereas that of R248Q is lower (indicating increased frustration) at $\chi \ = \ 0.21$ and that of the p53FG mutants is higher at $\chi \ = \ 0.32$. These results support our hypothesis by suggesting that inactivation of the R248Q protein results from an increased energetic barrier for the *S* → *R* transition, which is manifested in a 19% decrease in the similarity index. The increased activation rates of the p53FG mutant can be attributed to a decreased barrier, which is characterized by a 23% increase in the similarity index.

Similar results are obtained for the TUS and $\lambda$ repressor proteins. In the case of the TUS protein, both the K89A and the R198A mutants are characterized by decreased association rates (59) and accordingly show frustration indices decreased by 20% and 43%, respectively. The $\lambda$ repressor K4Q mutant is characterized by a significant reduction in activity (67,68) and a decrease in the similarity index of 28%, accordingly. In contrast, the E34K mutant, which is a secondary mutant that also contains the K4Q mutation, is characterized by a substantial increase in the similarity index from 0.13 for the K4Q mutant to 0.22 for the E34K mutant, which corresponds to a 22% increase with respect to the wild-type and is accordingly associated with an increased association rate. In all of the above cases, apart from the p53FG mutant, the mutations are associated with substitutions of charged residues that alter frustration by influencing the degree of overlap between specific protein–DNA contacts and the positively charged residues. In the case of the p53FG mutant, the variation in frustration is a result of modification to specific protein–DNA contacts.

## CONCLUSIONS

In this study, the facilitated diffusion model of DNA search by proteins for their target sites is revisited in order to consider explicitly not only the timescales for finding the target sites, but also the timescales for recognizing that site after it was identified. We show that these two processes of finding and binding the target sites are coupled via protein frustration between the non-specific and specific binding modes (i.e. the search, *S*, and recognition, *R*, states). Low frustration means that the non-specific and specific binding modes of the protein to the DNA are very similar, so recognition can occur relatively easily after the target is identified. Nonetheless, low frustration suggests hindered 1D diffusion because semi-specific protein–DNA interactions can be formed with higher probabilities. Accordingly, low frustration defines a low barrier for recognition but a more rugged protein–DNA energy landscape. High frustration, on the other hand, implies that a conformational change will be required during the transition from non-specific association with the specific complex. Indeed, in many protein–DNA complexes, either or both the protein and the DNA change conformation to affect specific binding (32–34). Our theory predicts that the magnitude of the conformational change depends on the similarity index. Consistently with this perspective, an NMR study of homeodomain HoxD9 that reported high similarity between the non-specific and specific binding modes (47,69) can be explained by the high similarity measure (i.e. low frustration) of this system (*χ* = 0.37). The experimentally estimated *D*_1D_ of HoxD9 (70) corresponds to an energetic ruggedness of ∼2*k*_B_*T*, again in agreement with low frustration. Lac repressor, on the other hand, is characterized by a lower similarity index (*χ* = 0.08) that explains the lower energetic ruggedness of <1 *k*_B_*T* for its 1D diffusion as was deduced experimentally (10). This high frustration may result not only in faster 1D diffusion but also in high transition barrier that may cause to unsuccessful recognition after finding the target site, as was indeed concluded experimentally for the lac repressor (8).

The concept of frustration between the *S* and *R* binding modes is utilized to successfully predict several experimentally measured phenomena. We show that the degree of frustration explains differences in the linear diffusion coefficients for sliding along DNA and thus provides a molecular interpretation for the experimentally reported barrier to sliding (10,60). In particular, we argue that architectural proteins slide along DNA with a smaller diffusion coefficient due to lower frustration. Furthermore, the concept of frustration has practical implications in predicting variations in recognition kinetics, particularly the effect of point mutations on recognition rates. Although mutations that change specific hydrogen bonds with the target DNA sites often affect the dissociation rates (i.e. *k*_off_), some mutations that involve positively charged residues are found to affect the association rate (i.e. *k*_on_). The latter can be rationalized as a change in the molecular frustration between the *S* and *R* states upon mutation. This also implies that the protein–DNA binding affinity can be modulated via *k*_on_ and not only via *k*_off_, as is often found. Our study explains the origin of the experimentally observed changes in association rate with salt concentration while the dissociation rates are hardly changed (59). Changing the energy of the specific complex (for example, by mutating the DNA target site) is expected to have a much greater effect on the dissociation rates than on the association effects, as was reported earlier for several proteins using comprehensive kinetic measurements (61).

Our study illustrates that there is a trade-off between the speed of sliding diffusion during the search process and the kinetics of the transition from the search mode to the recognition mode. Accordingly, our model suggests that the speed of sliding dynamics and the speed of recognition are tightly coupled. Most importantly, this trade-off is modulated by frustration between the non-specific and specific protein–DNA interactions and therefore there is an optimal degree of frustration that minimizes the total time for recognition (i.e. search plus recognition). The concept of frustration suggests that it is not only the search process that dictates the overall recognition time but also the energetic barrier for the specific complex. The magnitude of the trade-off depends on various parameters of the protein-DNA system such as the nature of the conformational change and the protein structures (e.g. existence of multi domains (14)). Finally, we show that our model is supported by experimental results, which find a strong correlation between the similarity index and the kinetic properties of protein–DNA recognition. Our model advances understanding and quantifies the relationship between protein characteristics and the facilitated diffusion mechanism and thus can be utilized to assist in the design of mutations to engineer and control the kinetic search process of DBPs.

## Supplementary Material

gkz308_Supplemental_File

## References

[1] Riggs A.D., Bourgeois S., Cohn M. The lac repressor-operator interaction. 3. Kinetic studies. J. Mol. Biol. 1970; 53:401–417. 4924006 10.1016/0022-2836(70)90074-4

[2] Berg O.G., Winter R.B., von Hippel P.H. Diffusion-driven mechanisms of protein translocation on nucleic acids. 1. Models and theory. Biochemistry. 1981; 20:6929–6948. 7317363 10.1021/bi00527a028

[3] von Hippel P.H., Berg O.G. Facilitated target location in biological systems. J. Biol. Chem. 1989; 264:675–678. 2642903

[4] Givaty O., Levy Y. Protein sliding along DNA: dynamics and structural characterization. J. Mol. Biol. 2009; 385:1087–1097. 19059266 10.1016/j.jmb.2008.11.016

[5] Bhattacherjee A., Krepel D., Levy Y. Coarse-grained models for studying protein diffusion along DNA. Wiley Interdiscip. Rev. Comput. Mol. Sci. 2016; 6:515–531.

[6] Marcovitz A., Levy Y. Sliding dynamics along DNA: a molecular perspective. RSC Biomolecular Sciences No. 24, Innovations in Biomolecular Modeling and Simulations. 2012; 2:236–262.

[7] Bauer M., Metzler R. Generalized facilitated diffusion model for DNA-binding proteins with search and recognition states. Biophys. J. 2012; 102:2321–2330. 22677385 10.1016/j.bpj.2012.04.008 PMC3353100

[8] Hammar P., Leroy P., Mahmutovic A., Marklund E.G., Berg O.G., Elf J. The lac repressor displays facilitated diffusion in living cells. Science. 2012; 336:1595–1598. 22723426 10.1126/science.1221648

[9] Slutsky M., Mirny L.A. Kinetics of protein-DNA interaction: facilitated target location in sequence-dependent potential. Biophys. J. 2004; 87:4021–4035. 15465864 10.1529/biophysj.104.050765 PMC1304911

[10] Blainey P.C., Luo G., Kou S.C., Mangel W.F., Verdine G.L., Bagchi B., Xie X.S. Nonspecifically bound proteins spin while diffusing along DNA. Nat. Struct. Mol. Biol. 2009; 16:1224–1229. 19898474 10.1038/nsmb.1716 PMC2889498

[11] Zhou H.X. Rapid search for specific sites on DNA through conformational switch of nonspecifically bound proteins. Proc. Natl. Acad. Sci. U.S.A. 2011; 108:8651–8656. 21543711 10.1073/pnas.1101555108 PMC3102365

[12] Krepel D., Levy Y. Protein diffusion along DNA: on the effect of roadblocks and crowders. J Phys a-Math Theor. 2016; 49:494003.

[13] Krepel D., Gomez D., Klumpp S., Levy Y. Mechanism of facilitated diffusion during a DNA search in crowded environments. J. Phys. Chem. B. 2016; 120:11113–11122. 27723976 10.1021/acs.jpcb.6b07813

[14] Zandarashvili L., Esadze A., Vuzman D., Kemme C.A., Levy Y., Iwahara J. Balancing between affinity and speed in target DNA search by zinc-finger proteins via modulation of dynamic conformational ensemble. Proc. Natl. Acad. Sci. U.S.A. 2015; 112:E5142–E5149. 26324943 10.1073/pnas.1507726112 PMC4577168

[15] Vuzman D., Levy Y. The “Monkey-Bar” mechanism for searching for the DNA target site: the molecular determinants. Israel J. Chem. 2014; 54:1374–1381.

[16] Vuzman D., Levy Y. Intrinsically disordered regions as affinity tuners in protein-DNA interactions. Mol. BioSyst. 2012; 8:45–57. 10.1039/c1mb05273j 21918774

[17] Vuzman D., Polonsky M., Levy Y. Facilitated DNA search by multidomain transcription factors: cross talk via a flexible linker. Biophys. J. 2010; 99:1202–1211. 20713004 10.1016/j.bpj.2010.06.007 PMC2920665

[18] Khazanov N., Marcovitz A., Levy Y. Asymmetric DNA-search dynamics by symmetric dimeric proteins. Biochemistry. 2013; 52:5335–5344. 23866074 10.1021/bi400357m

[19] Khazanov N., Levy Y. Sliding of p53 along DNA can be modulated by its oligomeric state and by cross-talks between its constituent domains. J. Mol. Biol. 2011; 408:335–355. 21338609 10.1016/j.jmb.2011.01.059

[20] Bhattacherjee A., Levy Y. Search by proteins for their DNA target site: 1. The effect of DNA conformation on protein sliding. Nucleic Acids Res. 2014; 42:12404–12414. 25324308 10.1093/nar/gku932 PMC4227778

[21] Bhattacherjee A., Levy Y. Search by proteins for their DNA target site: 2. The effect of DNA conformation on the dynamics of multidomain proteins. Nucleic Acids Res. 2014; 42:12415–12424. 25324311 10.1093/nar/gku933 PMC4227779

[22] Tafvizi A., Mirny L.A., van Oijen A.M. Dancing on DNA: kinetic aspects of search processes on DNA. Chemphyschem. 2011; 12:1481–1489. 21560221 10.1002/cphc.201100112 PMC4590286

[23] Mirny L., Slutsky M., Wunderlich Z., Tafvizi A., Leith J., Kosmrlj A. How a protein searches for its site on DNA: the mechanism of facilitated diffusion. J. Phys. A Math. Theor. 2009; 42:434013.

[24] Halford S.E., Marko J.F. How do site-specific DNA-binding proteins find their targets. Nucleic Acids Res. 2004; 32:3040–3052. 15178741 10.1093/nar/gkh624 PMC434431

[25] Wunderlich Z., Mirny L.A. Spatial effects on the speed and reliability of protein-DNA search. Nucleic Acids Res. 2008; 36:3570–3578. 18453629 10.1093/nar/gkn173 PMC2441786

[26] Halford S.E. An end to 40 years of mistakes in DNA-protein association kinetics. Biochem. Soc. Trans. 2009; 37:343–348. 19290859 10.1042/BST0370343

[27] Kolomeisky A. Physics of protein-DNA interactions: mechanisms of facilitated target search. Phys. Chem. Chem. Phys. 2011; 13:2088–2095. 21113556 10.1039/c0cp01966f

[28] Shvets A.A., Kochugaeva M.P., Kolomeisky A.B. Mechanisms of protein search for targets on DNA: theoretical insights. Molecules. 2018; 23:2106. 10.3390/molecules23092106 PMC6225296 30131459

[29] Sokolov I.M., Metzler R., Pant K., Williams M.C. Target search of N sliding proteins on a DNA. Biophys. J. 2005; 89:895–902. 15908574 10.1529/biophysj.104.057612 PMC1366639

[30] Brackley C.A., Cates M.E., Marenduzzo D. Intracellular facilitated diffusion: searchers, crowders, and blockers. Phys. Rev. Lett. 2013; 111:108101. 25166711 10.1103/PhysRevLett.111.108101

[31] Hu T., Grosberg A.Y., Shklovskii B.I. How proteins search for their specific sites on DNA: the role of DNA conformation. Biophys. J. 2006; 90:2731–2744. 16461402 10.1529/biophysj.105.078162 PMC1414577

[32] Viadiu H., Aggarwal A.K. Structure of BamHI bound to nonspecific DNA: a model for DNA sliding. Mol. Cell. 2000; 5:889–895. 10882125 10.1016/s1097-2765(00)80329-9

[33] Kalodimos C.G., Biris N., Bonvin A.M., Levandoski M.M., Guennuegues M., Boelens R., Kaptein R. Structure and flexibility adaptation in nonspecific and specific protein-DNA complexes. Science. 2004; 305:386–389. 15256668 10.1126/science.1097064

[34] von Hippel P.H. From ‘simple’ DNA-protein interactions to the macromolecular machines of gene expression. Annu. Rev. Biophys. Biomol. Struct. 2007; 36:79–105. 17477836 10.1146/annurev.biophys.34.040204.144521 PMC2660389

[35] Kochugaeva M.P., Shvets A.A., Kolomeisky A.B. How conformational dynamics influences the protein search for targets on DNA. J. Phys. A Math. Theor. 2016; 49:444004.

[36] Niranjani G., Murugan R. Generalized theory on the mechanism of site-specific DNA-protein interactions. J Stat Mech-Theory E. 2016; 2016:053501. 10.1088/1478-3975/13/4/046003 27434174

[37] Murugan R. Generalized theory of site-specific DNA-protein interactions. Phys. Rev. E. 2007; 76:011901. 10.1103/PhysRevE.76.011901 17677488

[38] Hu L.H., Grosberg A.Y., Bruinsma R. Are DNA transcription factor proteins Maxwellian Demons. Biophys. J. 2008; 95:1151–1156. 18456820 10.1529/biophysj.108.129825 PMC2479577

[39] Bauer M., Rasmussen E.S., Lomholt M.A., Metzler R. Real sequence effects on the search dynamics of transcription factors on DNA (vol 5, 10072, 2015). Sci. Rep. 2015; 5:10072. 26154484 10.1038/srep10072 PMC5507490

[40] Marcovitz A., Levy Y. Frustration in protein–DNA binding influences conformational switching and target search kinetics. Proc. Natl. Acad. Sci. U.S.A. 2011; 108:17957–17962. 22003125 10.1073/pnas.1109594108 PMC3207685

[41] Marcovitz A., Levy Y. Weak frustration regulates sliding and binding kinetics on rugged protein-DNA landscapes. J. Phys. Chem. B. 2013; 117:13005–13014. 23668488 10.1021/jp402296d

[42] Tafvizi A., Huang F., Fersht A.R., Mirny L.A., van Oijen A.M. A single-molecule characterization of p53 search on DNA. Proc. Natl. Acad. Sci. U.S.A. 2011; 108:563–568. 21178072 10.1073/pnas.1016020107 PMC3021058

[43] Zandarashvili L., Vuzman D., Esadze A., Takayama Y., Sahu D., Levy Y., Iwahara J. Asymmetrical roles of zinc fingers in dynamic DNA-scanning process by the inducible transcription factor Egr-1. Proc. Natl. Acad. Sci. U.S.A. 2012; 109:E1724–E1732. 22675124 10.1073/pnas.1121500109 PMC3387110

[44] Cuculis L., Abil Z., Zhao H.M., Schroeder C.M. Direct observation of TALE protein dynamics reveals a two-state search mechanism. Nat. Commun. 2015; 6:7277. 26027871 10.1038/ncomms8277 PMC4458887

[45] Cuculis L., Abil Z., Zhao H.M., Schroeder C.M. TALE proteins search DNA using a rotationally decoupled mechanism. Nat. Chem. Biol. 2016; 12:831–837. 27526029 10.1038/nchembio.2152

[46] von Hippel P.H. Biochemistry. Completing the view of transcriptional regulation. Science. 2004; 305:350–352. 15256661 10.1126/science.1101270

[47] Iwahara J., Clore G.M. Detecting transient intermediates in macromolecular binding by paramagnetic NMR. Nature. 2006; 440:1227–1230. 16642002 10.1038/nature04673

[48] Iwahara J., Zweckstetter M., Clore G.M. NMR structural and kinetic characterization of a homeodomain diffusing and hopping on nonspecific DNA. Proc. Natl. Acad. Sci. U.S.A. 2006; 103:15062–15067. 17008406 10.1073/pnas.0605868103 PMC1622777

[49] Ferreiro D.U., Komives E.A., Wolynes P.G. Frustration in biomolecules. Quarter. Rev. Biophys. 2014; 47:285–363. 10.1017/S0033583514000092 PMC4256721 25225856

[50] Slutsky M. Protein-DNA interaction, random walks and polymer statistics. 2005; MIT June 2005 https://dspace.mit.edu/handle/1721.1/32295?show=full.

[51] Bagchi B., Blainey P.C., Xie X.S. Diffusion constant of a nonspecifically bound protein undergoing curvilinear motion along DNA. J. Phys. Chem. B. 2008; 112:6282–6284. 18321088 10.1021/jp077568f

[52] Marcovitz A., Levy Y. Obstacles may facilitate and direct DNA search by proteins. Biophys. J. 2013; 104:2042–2050. 23663847 10.1016/j.bpj.2013.03.030 PMC3647173

[53] Benichou O., Chevalier C., Klafter J., Meyer B., Voituriez R. Geometry-controlled kinetics. Nat. Chem. 2010; 2:472–477. 20489716 10.1038/nchem.622

[54] Pulkkinen O., Metzler R. Distance matters: the impact of gene proximity in bacterial gene regulation. Phys Rev Lett. 2013; 110:198101. 23705743 10.1103/PhysRevLett.110.198101

[55] Di Stefano M., Rosa A., Belcastro V., di Bernardo D., Micheletti C. Colocalization of Coregulated Genes: A Steered Molecular Dynamics Study of Human Chromosome 19. PLoS Comput. Biol. 2013; 9:e1003019. 23555238 10.1371/journal.pcbi.1003019 PMC3610629

[56] Cencini M., Pigolotti S. Energetic funnel facilitates facilitated diffusion. Nucleic Acids Res. 2018; 46:558–567. 29216364 10.1093/nar/gkx1220 PMC5778461

[57] Grebenkov D., Metzler R., Oshanin G. Strong defocusing of molecular reaction timesresults from an interplay of geometry andreaction control. Commun. Chem. 2018; 1:96.

[58] Godec A., Metzler R. Universal proximity effect in target search kinetics in the few-encounter limit. Phys. Rev. X. 2016; 6:041037.

[59] Neylon C., Brown S.E., Kralicek A.V., Miles C.S., Love C.A., Dixon N.E. Interaction of the Escherichia coli replication terminator protein (Tus) with DNA: A model derived from DNA-binding studies of mutant proteins by surface plasmon resonance. Biochemistry. 2000; 39:11989–11999. 11009613 10.1021/bi001174w

[60] Kamagata K., Mano E., Ouchi K., Kanbayashi S., Johnson R.C. High free-energy barrier of 1D diffusion along DNA by architectural DNA-binding proteins. J. Mol. Biol. 2018; 430:655–667. 29307468 10.1016/j.jmb.2018.01.001 PMC7594881

[61] Geertz M., Shore D., Maerkl S.J. Massively parallel measurements of molecular interaction kinetics on a microfluidic platform. Proc. Natl. Acad. Sci. U.S.A. 2012; 109:16540–16545. 23012409 10.1073/pnas.1206011109 PMC3478601

[62] Maerkl S.J., Quake S.R. A systems approach to measuring the binding energy landscapes of transcription factors. Science. 2007; 315:233–237. 17218526 10.1126/science.1131007

[63] Itoh Y., Murata A., Sakamoto S., Nanatani K., Wada T., Takahashi S., Kamagata K. Activation of p53 facilitates the target search in DNA by enhancing the target recognition probability. J. Mol. Biol. 2016; 428:2916–2930. 27291286 10.1016/j.jmb.2016.06.001

[64] Ory K., Legros Y., Auguin C., Soussi T. Analysis of the most representative tumor-derived p53 mutants reveals that changes in protein conformation are not correlated with loss of transactivation or inhibition of cell-proliferation. EMBO J. 1994; 13:3496–3504. 8062826 10.1002/j.1460-2075.1994.tb06656.x PMC395253

[65] Emamzadah S., Tropia L., Vincenti I., Falquet B., Halazonetis T.D. Reversal of the DNA-Binding-Induced Loop L1 Conformational Switch in an Engineered Human p53 Protein. J. Mol. Biol. 2014; 426:936–944. 24374182 10.1016/j.jmb.2013.12.020

[66] Petty T.J., Emamzadah S., Costantino L., Petkova I., Stavridi E.S., Saven J.G., Vauthey E., Halazonetis T.D. An induced fit mechanism regulates p53 DNA binding kinetics to confer sequence specificity. EMBO J. 2011; 30:2167–2176. 21522129 10.1038/emboj.2011.127 PMC3117648

[67] Hecht M.H., Nelson H.C.M., Sauer R.T. Mutations in lambda-repressors amino-terminal domain - implications for protein stability and DNA-binding. Proc. Natl. Acad. Sci. U.S.A. 1983; 80:2676–2680. 6221342 10.1073/pnas.80.9.2676 PMC393890

[68] Nelson H.C.M., Sauer R.T. Lambda repressor mutations that increase the affinity and specificity of operator binding. Cell. 1985; 42:549–558. 3161621 10.1016/0092-8674(85)90112-6

[69] Iwahara J., Clore G.M. Direct observation of enhanced translocation of a homeodomain between DNA cognate sites by NMR exchange spectroscopy. J .Am. Chem. Soc. 2006; 128:404–405. 16402815 10.1021/ja056786o

[70] Sahu D., Iwahara J. Discrete-state kinetics model for NMR-based analysis of protein translocation on DNA at equilibrium. J. Phys. Chem. B. 2017; 121:9548–9556. 28922916 10.1021/acs.jpcb.7b07779 PMC5661886

