# Supplementary material for: Quantifying the two-state facilitated diffusion model of protein–DNA interactions
Source: Nucleic Acids Res. 2019 May 2;47(11):5530–8. doi: 10.1093/nar/gkz308 (PMC6582340; doi:10.1093/nar/gkz308)
Supplement: gkz308_Supplemental_File [file gkz308_supplemental_file.docx]

**Quantifying the two-state facilitated diffusion model of protein­–DNA interactions**

Itai Leven and Yaakov Levy

**Supplementary Information**

To evaluate the relationship between the barrier height for the *S → R* transition and the similarity index χ, we performed coarse-grained simulations of transition from the nonspecific binding mode to the specific binding modes to six different proteins whose χ value ranges between 0.02-0.42. Each protein was first simulated using the model we applied to study sliding of proteins along DNA. In this model, only the electrostatic interactions are included. Using this model, the nonspecific interaction of each DNA-binding protein with DNA is characterized. The binding mode that is governed by only electrostatic interactions mimics the *S* state. To introduce the *R* state in the model, the specific interactions found in the X-ray structure of each protein with DNA were added to the model as Lennard-Jones interactions, all with energetic contribution of ε=1. We then started simulations in which the protein starts at the *S* state and we measured the time it takes to move to the *R* state (i.e., the time in which all the Lennard-Jones interactions are formed). The relationship between the kinetics of *S → R* and χ follows an exponential function (see Fig. S1) and accordingly the free energy barrier for the *S → R* transition linearly depends on χ.

Figure S1
